# Complete Genome Sequence of *Lactobacillus acidophilus* FSI4, Isolated from Yogurt

**DOI:** 10.1128/genomeA.00166-15

**Published:** 2015-04-09

**Authors:** Oleg Iartchouk, Sergei Kozyavkin, Valeri Karamychev, Alexei Slesarev

**Affiliations:** a Novartis Institutes for BioMedical Research (NIBR), Cambridge, Massachusetts, USA; b Fidelity Systems, Inc., Gaithersburg, Maryland, USA; c Winogradsky Institute of Microbiology, RAS, Moscow, Russia

## Abstract

A new *Lactobacillus acidophilus* strain, FSI4, isolated from yogurt, was isolated and sequenced in our laboratory. Our data, although supportive of previous conclusions regarding the remarkable stability of *L. acidophilus* species, indicate accumulating mutations in commercial *L. acidophilus* strains that warrant further study of the effect of damaged genes on the competitiveness of these bacteria in gut microbiota.

## GENOME ANNOUNCEMENT

*Lactobacillus acidophilus* is a member of the Gram-positive bacterial group capable of producing lactic acid (1–3). This capability has resulted in their use in the dairy industry, with *L. acidophilus* being the most widely commercially distributed culture (4). *L. acidophilus* is one of the first colonizers of the digestive tract of newborns, and its competitive advantage over other bacteria plays an important role in the healthy development of infants (5, 6). There are reports of multiple draft genome sequences of *L. acidophilus* strains (4, 7), but only two complete genome sequences are available (8, 9).

The complete genome sequence of *L. acidophilus* FSI4 was determined by the combined use of the Illumina and Sanger sequencing platforms. Illumina GAIIx paired-end reads (153× coverage) were assembled with Velvet (10). Four rRNA operons and 4 types of long insertion sequence (IS) elements with pairwise similarities in the range of 96.3 to 100% were identified. Their positions in the chromosome were verified by PCR (rRNA genes) and by direct Sanger sequencing from genomic DNA (11, 12) through the IS elements, with the use of trimming technique. This technique involves the hybridization of the Sanger sequencing fragments with appropriate biotinylated complementary oligonucleotides 600 to 900 bases downstream of the primer, followed by trimming the duplex with a 4-base-cutter restriction endonuclease and running the trimmed fragments separately on a capillary sequencer.

The *L. acidophilus* FSI4 chromosome encodes 1,759 proteins, 112 RNAs, 54 riboswitches and leaders, and 98 pseudogenes. Alignment of the *L. acidophilus* genomes demonstrates that strains FSI4, NCFM, and LA-14 are extremely similar, with all genes being syntenic across all three genomes. We have found a single 9-nucleotide deletion in the NADH dehydrogenase gene in the FSI4 strain, compared to the NCFM and LA-14 strains, which results in 3 amino acid in-frame deletions in the corresponding protein. For a further comparison of the FSI4 and NCFM genomes, we first corrected sequencing errors in the NCFM genome with the Illumina data obtained by Bull et al. (4) and then analyzed the remaining differences that happened to be limited to 9 single-nucleotide substitutions. We also noticed that the majority of the identified indels and single-nucleotide polymorphisms (SNPs) (40 total) in the LA-14 genome occur in the homopolymer stretches, pointing to the possibility that they might be errors resulting from the 454 sequencing used in the LA-14 genome project (9, 13). Finally, there is a 54-bp variable region in one of the 1,522-bp IS elements of the FSI4 genome that is different in the corresponding copies of the 1,522-bp IS elements of the NCFM and LA-14 genomes.

Overall, our results, along with previous data, prove that commercial *L. acidophilus* genomes have remarkable macrostability. On the other hand, our highly accurate sequencing data reveal the presence of multiple disrupted genes in the genomes in question. We showed previously that gene disruption during the cultivation of *Bifidobacterium longum* may reduce the competitiveness of probiotics in the gut (14). However, further studies are required to clarify the effect of gene restoration on the competitive advantage of probiotics in the gut microbiota.

### Nucleotide sequence accession numbers. 

The complete genome sequence of *L. acidophilus* FSI4 has been deposited in GenBank under the accession no. CP010432. The 100-bp Illumina HiSeq 2000 reads used for error correction of the *L. acidophilus* NCFM genome (accession no. CP000033) are available in the NCBI sequence read archive (SRA) under accession no. ERR386044.
